# Perceptual organization of auditory streaming-task relies on neural entrainment of the stimulus-presentation rate: MEG evidence

**DOI:** 10.1186/1471-2202-14-120

**Published:** 2013-10-12

**Authors:** Ivan Chakalov, Rossitza Draganova, Andreas Wollbrink, Hubert Preissl, Christo Pantev

**Affiliations:** 1 Institute for Biomagnetism and Biosignalanalysis, University of Münster, Malmedyweg 15, 48149 Münster, Germany; 2 MEG-Center, Eberhard-Karls-University Tübingen, Otfried-Müller-Straße 47, 72076 Tübingen, Germany

**Keywords:** MEG, Time-frequency spectrum, Auditory scene analysis, Task-driven entrainment

## Abstract

**Background:**

Humans are able to extract regularities from complex auditory scenes in order to form perceptually meaningful elements. It has been shown previously that this process depends critically on both the temporal integration of the sensory input over time and the degree of frequency separation between concurrent sound sources. Our goal was to examine the relationship between these two aspects by means of magnetoencephalography (MEG). To achieve this aim, we combined time-frequency analysis on a sensor space level with source analysis. Our paradigm consisted of asymmetric ABA-tone triplets wherein the B-tones were presented temporally closer to the first A-tones, providing different tempi within the same sequence. Participants attended to the slowest B-rhythm whilst the frequency separation between tones was manipulated (0-, 2-, 4- and 10-semitones).

**Results:**

The results revealed that the asymmetric ABA-triplets spontaneously elicited periodic-sustained responses corresponding to the temporal distribution of the A-B and B-A tone intervals in all conditions. Moreover, when attending to the B-tones, the neural representations of the A- and B-streams were both detectable in the scenarios which allow perceptual streaming (2-, 4- and 10-semitones). Alongside this, the steady-state responses tuned to the presentation of the B-tones enhanced significantly with increase of the frequency separation between tones. However, the strength of the B-tones related steady-state responses dominated the strength of the A-tones responses in the 10-semitones condition. Conversely, the representation of the A-tones dominated the B-tones in the cases of 2- and 4-semitones conditions, in which a greater effort was required for completing the task. Additionally, the P1 evoked fields’ component following the B-tones increased in magnitude with the increase of inter-tonal frequency difference.

**Conclusions:**

The enhancement of the evoked fields in the source space, along with the B-tones related activity of the time-frequency results, likely reflect the selective enhancement of the attended B-stream. The results also suggested a dissimilar efficiency of the temporal integration of separate streams depending on the degree of frequency separation between the sounds. Overall, the present findings suggest that the neural effects of auditory streaming could be directly captured in the time-frequency spectrum at the sensor-space level.

## Background

Humans are able to organize perceptually meaningful elements from the mixture of competing sounds in the environment. Albert Bregman has described this phenomenon in the well-known framework of Auditory Scene Analysis [1]. The vast majority of researchers interpret the streaming effect in terms of tonotopic organization of the auditory system [2-17]. According to this interpretation, frequency-distant sounds are processed into distinct neural populations and therefore heard as separate streams, and frequency adjacent sounds are processed in neighboring neural channels leading to their perceptual integration into one unified auditory object. However, it has recently been shown that streams compiled from frequency-remote tones can no longer be heard as distinct sound streams if the tones are presented synchronously rather than successively, despite the enhanced neural activity [18,19]. Therefore, the tonotopic organization per se does not explain completely the perception of streaming [18,19]. The formation of different auditory streams requires temporal integration of sound input over time [20,21].

On the other hand, numerous studies have evaluated the use of the event-related oscillations to indicate the process of sensory integration in the brain [22-32]. Lins & Picton, for instance, found that multiple auditory stimuli evoke steady-state activity following their repetition rate [23]. More recent research showed that selective attention could modulate the steady-state responses within the auditory system [33,34] and between different sensory modalities (e.g. between visual and auditory [35,36]). In addition, an electroencephalographic study by Nozaradan and colleagues demonstrated that musical beat could elicit a steady-state response tuned to the beat frequency and, furthermore, that binary and ternary metric interpretation of this beat evoked frequencies tuned to the corresponding imagery meter [37].

With regard to auditory stream segregation, it has been demonstrated that attending to a certain rhythm enhances the magnitude of the steady-state response corresponding to its presentation rate [20]. Nevertheless, these authors investigated the neural representation of two recurring sound sequences separated by a relatively large inter-tonal frequency difference [20] paradigm which primarily produces two auditory streams. An intriguing question, therefore, is how different degrees of inter-tonal frequency separation between the sounds, and respectively different perceptual states, affect the spectral distribution of one and the same polyrhythmic structure.

The present study used magnetoencephalography (MEG) to address this question. Experimental block of three subsequent parts was carried out in which the rhythmic elements were kept constant but the frequency separation between the sounds was systematically manipulated. Specifically, in the first two parts a variation of the standard ABA-triplet paradigm was used [38], wherein the B-tones were set temporally closer to the first A-tones than to the second A-tones, forming dissimilar rhythms within the same sequence. In the first part, two extreme frequency separations between A- and B-tones, which are respectively known to form the perception of one stream vs. two streams [38], were contrasted (0- vs. 10-semitones). In the second part, small (2-semitones) vs. intermediate (4-semitones) frequency separations were opposed, ensuring bi-stable perception [38]. In order to keep sustained attention during all these conditions, the participants were asked to follow the presentation of the B-tones and indicate the switching of their perception from the ABA-rhythm to two separate A- and B-tone streams by pressing a button. A combination of time-frequency analysis on a sensor space level and source analysis were used to analyze the results. We anticipated that in the first part our analysis would capture activity corresponding to the temporal distribution of the A-B and B-A-tone intervals of the ABA-triplets in the single-stream condition (0-semitones). In the streaming condition (10-semitones) presentation frequencies of clear B-tones and A-tones were expected. In the second part (2- and 4-semitones) all presentation rates were expected in the spectrum (responses, relevant to presentation rates of both B- and A-tones and to the asymmetric ABA-triplets). Therefore, the present design provides a complementary model for investigation of stream integration versus stream segregation by varying the inter-tonal frequency separation between the A- and B-tones in a classical streaming task. Accordingly, based on the different presentation rates we could access different perceptual states (integrated versus segregated) by capturing the corresponding frequencies to these different rates in the time-frequency spectrum. Additionally, for the first two parts, source waveforms synchronized to the B-tones of the ABA-structure were extracted, in order to be associated with the time-frequency data.

The third part consisted of a sequence compiled from two independent, simultaneously presented A- and B-tone streams (not as ABA-triplet). In this part the participants were not required to follow any of the rhythms. The A- and B-tones of the two presented sequences appeared always at different temporal relation to each other. Conversely, the temporal distributions between the A- and the B-tones per se were always regular. The auditory system prefers to organize separate streams based on regularities such as pitch [2] and regular temporal arrangement [39,40]. On the other hand, it has recently been demonstrated that streaming can occur without any difference in the fundamental frequency [41] and an integrated percept can occur with irregular arrangements [42]. Therefore, if the auditory system is capable of integrating simultaneously the A- and B-tones into separate streams, based on their regular presentation rates and identical tone-frequencies, then two steady-state responses related to their frequency distribution would be captured in the spectrum.

## Methods

### Participants

Fourteenth right-handed participants (5 males), aged between 22 and 30 years, participated in this study. None of them had a history of otological or neurological disorders. A normal audiological status was verified by pure-tone audiometry in terms of air conduction hearing thresholds less than 10 dB. Pure-tone thresholds were measured for octave frequency from 250 to 4000 Hz. All participants gave a written, informed consent in accordance with the Declaration of Helsinki. The study protocol was approved by the Ethics Commission of the University of Münster, Germany (WWU-Muenster).

### Experimental procedures

The experiments were organized as experimental block of three following parts. 5 min. silent gaps divided the parts. Non-regular ABA-triplet sequences were used in the first two parts. The stimuli were sinusoidal tone-pips of 25 ms duration, including 10 ms rise and decay times. The loudness of the stimuli was set to 60 dB above the individual hearing thresholds. The duration of each trial was 5 s. The inter trial interval (ITI) was set to 3 s and the total recording time of one experimental part was 10.6 minutes.

**Figure 1:**
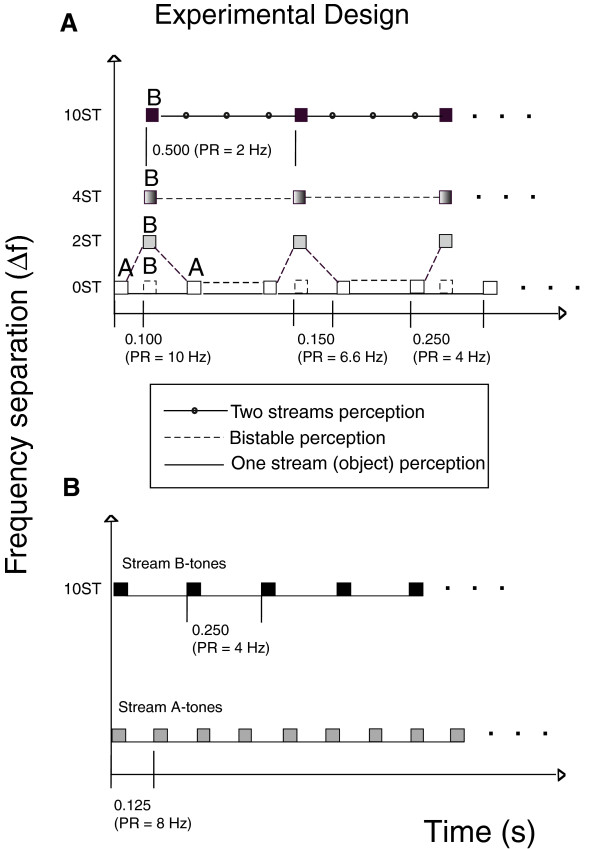
**Experimental design.** The conditions of the three experimental parts are presented as relative Frequency against relative Time. **(A) *Parts 1 and 2***. The Sound Onset Asynchrony (SOA) between the successive A-tones was 250 ms, corresponding to a presentation rate of 4 Hz. The SOA between the successive B-tones was 500 ms, corresponding to an SOA of 2 Hz. The SOAs linking the irregular ABA structure were set to 100 ms (A-B segment) and 150 ms (B-A segment), corresponding to 10 and 6.6 Hz rates, respectively. Four different degrees of Δf were opposed in part 1 (0 vs. 10-semitones) and part 2 (2 vs. 4-semitones). **(B) *Part 3***. Independent presentation of A- and B-tone streams with Δf of 10-semitones. The presentation rates of the A- and B-tones corresponded to 8 Hz (SOA = 125 ms) and 4 Hz (SOA = 250 ms), respectively.

In the first part, the single auditory-stream (object) condition was compared with the streaming-condition (0 vs. 10-semitones ∆f [frequency difference]), with the exact ordering of these conditions randomized. The second part compared small (2-semitones) versus intermediate (4-semitones) ∆f, in the same way. In all conditions, the frequency of tone A was 500 Hz, but the B-tones were 500 Hz (single-object condition), 561 Hz (small ∆f), 630 Hz (intermediate ∆f) and 891 Hz for the streaming condition, Figure 1A. In the first two parts, the Sound Onset Asynchrony (SOA) between the successive A-tones was always 250 ms, which corresponds to a presentation rate of 4 Hz. The SOA between the successive B-tones was 500 ms, which corresponds to 2 Hz presentation rate. The SOA linking the first A-tone and the next B-tone of the asymmetric ABA-structure was 100 ms, thus the SOA between the B-tone and the second A-tone was 150 ms, which corresponds to presentation rates of 10 Hz and 6.6 Hz, respectively. The SOA between the ABA- triplets was also set to 250 ms (4 Hz), Figure 1A. In each experimental part, 80 trials were presented: 40 trials of each condition. The presentation order of the first and second parts was counterbalanced across subjects.

The asymmetric ABA-triplet paradigm used in this study allows two competing perceptual states (integrated vs. segregated). In order to provide an objective estimate of stream integration versus stream segregation we manipulate the ∆f between the A- and B-tones whilst keeping sustained attention. Therefore, before the main experiment, the participants were exercised with 10 trials of each condition that allows perceptual streaming (2-, 4- and 10-semitones) from parts 1 and 2 in order to segregate the asymmetric ABA- structure into separate A- and B-streams at the very first moment and to keep the perception as stable as possible. Accordingly, the participants were instructed to focus on the B-tones sequence (in all conditions) and to indicate if their perception switched from the repeated ABA-objects to two segregated B- and A-tone streams by pressing a mouse button after the presentation of each trial. However, we were not interested in the overall level of performance of this task but in maintaining sustained attention. Moreover, 10 of the present 14 participants took part in our previous research in which all of them were able to hear two streams in case of 2-, 4- and 10-semitones conditions [43]. The 0-semitones condition is assumed to be always heard as one object.

In the third part, two sequences of independent A- and B-sound-streams (instead of the triplet structure) were presented simultaneously for 5 seconds with an ITI of 3 seconds (80 trials for 15 minutes). The presentation rate of the A-tones, therefore, corresponded to 8 Hz (SOA = 125 ms) and the B-tones to 4 Hz (SOA = 250 ms). The frequency separation was set each time to 10-semitones (tone A = 500 Hz;-tone B = 891 Hz), Figure 1B. The subjects were not supposed to pay attention to the stimuli and, instead, watched a silent movie of their choice. However, before the main experiment all of the participants had reported that they can hear two streams in that condition. In additional five-minute part, the spontaneous brain activity was recorded in order to distinguish between the spectral power that corresponds to the expected target frequencies in the third part and the resting brain-state [44]. During the resting state recordings the same experimental design as in part 3 was presented to the participants, however, without audio output to the MEG-room. In that way the same conditions’ triggers were available for further epochnig.

### MEG data acquisition

The MEG recording was performed using a 275-channel whole-head system (Omega2005, VSM-Medtech, Port Coquitlam, BC, Canada), sampled at 600 Hz. The participants were seated comfortably in an upright position. The sensors were configured as first-order gradiometers with a baseline of 50 mm. In addition to the MEG, the electrooculogram (EOG) was recorded for subsequent artifact rejection. The participants’ head positions were determined at the beginning and at the end of each recording block by means of 3 localization coils fixed to the nasion and the entrances of both ear canals. Alertness and compliance were verified by video monitoring. The acoustic stimuli were delivered through a nonmagnetic and echo-free acoustic transmission system (VSM-Medtech, Port Coquitlam, BC, Canada) to silicon earpieces placed into the ear canals.

#### Time*-*frequency analysis

In this study we examined whether sequential auditory scene analysis relies on brain oscillations entrained to the stimulus presentation rates. For that purpose we investigated the hypothesized brain frequency oscillations by means of time-frequency analysis. The following MEG processing steps were performed using Matlab-2011a (The MathWorks, Natick, MA, USA) and the FieldTrip toolbox (http://www.ru.nl/neuroimaging/fieldtrip). Before starting the analyses, the continuous data were separated into epochs of 6 s (1 s before and 5 s after the onset of the trials). Epochs containing signals larger than 3 pT were considered as artifact-contaminated and excluded from the analysis. In the present study time-frequency representation of power was calculated based on Fourier analysis using a sliding (short time) window approach (mtmconvol). In order to reduce spectral leakage and to control the frequency smoothing prior to power calculation the data were multiplied with a single taper (Hanning Window). The length of the sliding window was set to a fixed number of periods resulting in shorter time windows with increasing frequency. To compensate the expense of frequency smoothing for higher frequencies whilst keeping a constant time window (from −1 to 5 s) we chose to independently analyze the data for two separate frequency ranges: one from 1 to 6 Hz and another one from 6 to 14 Hz. Frequency-dependent time window of 2 cycles was used to calculate the activity of the first range (from 1 to 6 Hz) and frequency-dependent time window of 13 cycles to calculate the second range (from 6 to 14 Hz). For each range the time-frequency power representation was calculated using a frequency resolution of 0.67 Hz and a temporal resolution of 50 ms.

**Figure 2:**
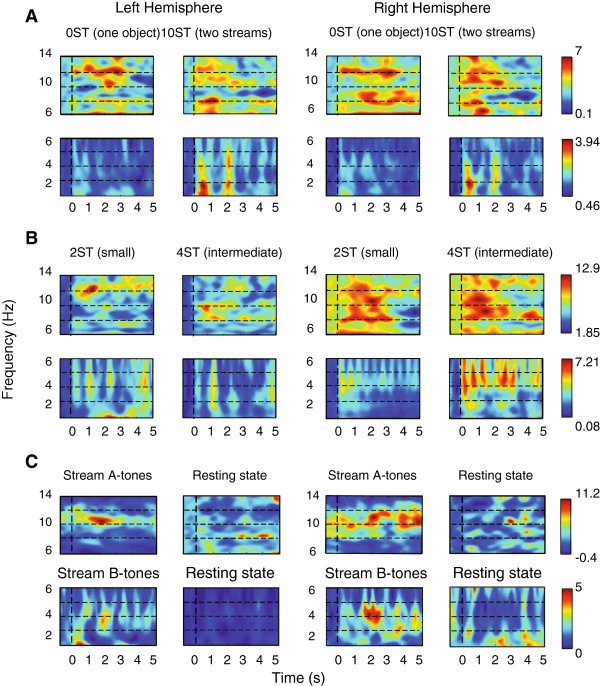
**Group level time-frequency plots.** The data were averaged across all trials and pre-selected MEG channels separately for the Left (LH) and Right (RH) Hemispheres. **(A) *Part 1***. Non-streaming vs. streaming conditions are presented independently for LH and RH. Upper plots represent the frequency range from 6 to 14 Hz; lower plots the range from 1 to 6 Hz. **(B) *Part 2***. Small vs. intermediate ∆f conditions are presented in the same way as part 1. **(C) *Part 3****.* The plots show simultaneous presentation of independent A- and B-sound streams and the relevant resting brain-state. The two frequency ranges; from 6 to 14 Hz (upper plots) and from 1 to 6 Hz (lower plots) are presented separately for the LH and RH. Color bars represent the relative change of the spectral power. The strength of the signal is color-coded: high strength is denoted with red and low strength with blue.

The topographic maps were also analyzed across the conditions depicting the averaged time course of each stimulus condition and each frequency range (see Additional file 1A), although the goal of the present study was not to focus on concrete brain structures. Based on the prior assumption of different hemispheric specialization in streaming [43] and the obtained dissimilar distribution of the grand averaged topography maps, the magnitudes of the proposed frequency ranges were analyzed separately for the left and right hemispheric channels (cf. Figure 2). The external frontal channels (above the supra-orbital ridges) and the EOG-channels were excluded from further analyses in order to additionally diminish the effects of eye blinks and frontal muscle activities. The most occipital channels were less active related to the other channels and therefore excluded from further analyses. At the end, 70 channels of each hemisphere were analyzed. The large number of channels used minimized the side effects of possible individual channel deviation. It should be noted here, that in order to avoid canceling the activities locked to stimulus rate in case of phase difference, the time-frequency representations were calculated for every trial of each individual (40 trials per condition) before averaging the data across the different conditions and participants. Therefore, we expected to capture the activity of approximately 2 Hz (B-tones presentation rate) and 4 Hz (A-tones presentation rate) that corresponds to streaming in the cases of the 10-, 4- and 2-semitones conditions (parts 1 and 2) into the first frequency range. In contrast, the activity reflecting the processing of A-B (10 Hz) and B-A (6.6 Hz) time intervals of the unified ABA objects was expected into the second frequency range. The same frequency ranges were analyzed for the third part and the resting state measurements, in order to capture the steady-state activity corresponding to the independent presentation rates of A- and B-tone streams and the relevant spontaneous activity at 8 Hz and 4 Hz. The time-frequency analyzed epochs were then averaged for each condition (40 epochs per condition in part 1 and part 2 and 80 epochs in part 3) across the channels. This was done independently for the left and right hemispheric channels. In order to extract unrelated outstanding noise, such as spontaneous brain activity, baseline-normalization was applied in terms of Relative Change of Spectral Power (*RCSP*). The *RCSP* expresses, for each frequency, the relative increase or decrease of the raw power values with respect to the power in the baseline interval. Thus, if *Pa* is the spectral power of the post-trigger time-period (from 0 to 5 s) and *Pb* is the spectral power of the pre-trigger period (from −1 to 0 s) the *RSCP* value is calculated as:

$$\mathrm{RCSP}=\left(\mathrm{Pa}-\mathrm{Pb}\right)/\mathrm{Pb}.$$

In addition, the analyzed mean epochs of each condition were averaged across all the participants with the intention of presenting the group-averaged effects. This procedure provided the grand average *RCSP* values, separately for the two frequency ranges (from 1 to 6 Hz and from 6 to 14 Hz), in the time window from −1 to 5 s, for each condition and hemisphere. It has been shown that the formation of different auditory streams needs a variable amount of time to build-up [16] therefore averaging across the participants could lead to cancelation of some effects based on the dissimilar individual percept over time. Thus, time-frequency responses of one participant were additionally analyzed (Additional file 1B).

Before entering statistical analysis, the mean *RCSP* values in the time window from 0 to 5 s post-trigger period were collected for each participant, condition and hemisphere in the following way: (1) for parts 1 and 2, the activity was extracted and averaged between 1.5 and 2.5 Hz and between 3.5 and 4.5 Hz, looking for the 2 Hz B-tones and the 4 Hz A-tones related steady-state evoked activity; (2) the activity was also averaged between 5.5 and 8 Hz and between 9 and 11.5 Hz, to identify frequencies of approximately 6.6 Hz and 10 Hz corresponding to the distribution of the tone intervals in the asymmetric ABA-triplets; (3) for part 3, the frequency bands between 3.5 and 4.5 Hz and between 7 and 9.5 Hz were used, with the expectation of finding 4 Hz B-tones and 8 Hz A-tones related steady-state evoked activity. The mean *RCSP* values of the resting state measurements between 3.5 and 4.5 Hz and between 7 and 9.5 Hz were also averaged from the additional resting-state part, in order to compare them with the relevant activity derived from part 3. The calculated mean values of the baseline corrected spectral power for each target frequency were then entered into statistical analysis.

To investigate whether the activity related to the distribution of the ABA-structure depends on the activity related to separate perception of A- and B-tone-streams (parts 1 and 2), the mean spectral power values of the different target frequencies across the different conditions were entered into 4 × 4 repeated-measure ANOVA using within-subject factors Target Frequency (10, 6.6, 4 and 2 Hz) and Condition (0, 2, 4 and 10-semitones). Thereafter, the mean spectral power of each target frequency from part 1 and part 2 were separately entered into a 2 × 4 repeated-measures ANOVAs, using within-subject factors Hemisphere (left, right) and Conditions (0-, 2-, 4- and 10-semitones). Thus, we were also able to explore how the spectral power of identical target frequencies changes across conditions, as well as their effects between the hemispheres.

Additionally, the mean values of the baseline corrected spectral power from part 3 were compared with the relevant mean values of spontaneous activity derived from the resting state-part (approx. 8 Hz and 4 Hz). Therefore, a separate 2 × 4 model ANOVA was applied here using within-subject factors Hemisphere (left, right) and Activity (spontaneous activity at approx. 8 Hz and 4 Hz and evoked activity at approx. 8 Hz and 4 Hz).

When significant, *post hoc* pairwise comparisons were performed using paired-samples *t-*tests. The alpha level was set at 0.05 and Bonferroni correction was applied in all analyses.

#### Analysis of source waveform data

The analysis was performed using the BESA software package (BESA GmbH, Version 5.7.3) and Matlab-2011a (The MathWorks, Natick, MA, USA), waveform-toolbox. Before starting the preprocessing procedure, the data were high pass-filtered with the lowest frequency limit of 1 Hz. The data were separated into epochs corresponding to the B-tones of the ABA-triplets, starting 50 ms before and ending 400 ms after the B-tone onset. Epochs containing signals larger than 3 pT were considered as artifacts and excluded from further analysis. Before averaging, the response signals were low pass filtered at 30 Hz. Each different condition was then averaged, in order to achieve the best signal-to-noise ratio. These procedures were performed only for the first two parts. The data of part 3 could not be epoched because no baseline could be derived as a result of the independent presentation of A- and B-tone streams.

The signal space projection technique [45] was used for the analysis of the MEG data. The interval used for the ECD fit (~30 ms) was placed around the local maximum of the N1 component of the AEF. The N1 dipolar sources evoked by B-tones of the ABA-triplets were less variable across conditions compared to the P1, P2 or N2 sources and, thus, provided a better signal-to-noise ratio. Each N1 dipole parameter was represented by the average of all data points (30 ms interval) around the maximum of the Global Field Power of the magnetic field calculated across the respective subsets of channels. Thereafter, the source space projection method was applied to calculate the components of the transient evoked response (P1, N1, P2 and etc.) [45]. For each participant and condition, two ECDs (one in each hemisphere) were determined by their dipole moment, orientation and spatial coordinates (a goodness of fit larger than 90% was imposed), a technique justified by other authors (e.g. see [29,46-48]).

A time window of 30 ms was placed around the individual peaks of P1 and N1 of the calculated AEFs in order to collect their amplitudes and latencies for further statistical analysis. Two participants in which the expected N1-responses could not be fitted into two dipoles were excluded from further analysis hence the responses of eleven participants were entered into statistics. The averaged amplitudes and latencies of P1 and N1 components at the 30 ms interval were then entered into repeated measures ANOVA with the within-subject factors Hemisphere (Left, Right) and Conditions (0-, 2-, 4- and 10-semitones). When significant, *post hoc* pairwise comparisons were performed using paired-samples *t-*tests. Bonferroni correction was applied for all analyses.

## Results

### Time-frequency data

#### Interactions between the spectral distribution of the ABA-structure and A- and B-streams

The polyrhythmic structure used in the present study consisted only of two tones (A and B) organized as an asymmetric ABA-triplet. These two tones could form different rhythms, depending on the listener’s current perceptual state [49]. This perceptual state is directly influenced by the inter-tonal frequency separation between the A- and B-tones. Therefore, when the perception is integrated as an ABA-stream, one would expect to capture the corresponding presentation frequencies into the spectrum. Conversely, in case of segregation (streaming), one should be able to capture, separately, the temporal distribution of the A- and B-streams in the spectrum. Hence, by varying the inter-tonal frequency separations, we expected to access integrated versus segregated percepts in the time-frequency spectrum.

As shown at the group data-plots (Figure 2AB), the presentation rate of the B-tones of the ABA-triplet sequences elicited a clear increase of the spectral power at about 2 Hz in the streaming (10-semitones) and in the intermediate frequency separation (4-semitones) conditions from the first and second parts. Additionally, the A-tones presentation elicited a steady-state like activity at about 4 Hz in the intermediate (4-semitones) and small frequency separation (2-semitones) conditions. The asymmetric ABA-objects induced enhanced activity in all four conditions (parts 1 and 2) at approximately 10 Hz and 6 Hz (Figure 2AB).

The ANOVAs revealed significant interaction between the spectral distribution of the ABA-objects and those of the separated A- and B-streams on the basis of the different frequency separation between the competing tones (main effect Conditions [*F*(3,39) = 5.335, *p < .001*], Target Frequency [*F*(3,39) = 18.550, *p < .001*] and interaction Conditions x Target Frequency [*F*(9,117) = 2.217, *p < .05*]).

*Post hoc* pairwise comparisons showed that the mean spectral power corresponding to separate perception of A- and B-streams has lower amplitude compared to the activity related to the distribution of the ABA-triplets in the conditions of small inter-tonal frequency separation. In particular, the B-tones related activity (2 Hz) of the non-streaming scenario (0-semitones) was significantly lower compared to spectral power of the A-B (approx. 10 Hz) and B-A (approx. 6 Hz) tone intervals, *t*(13) = −3.434, *p* < .*001* and *t*(13) = −5.620, *p* < .*001*. The A-tones (4 Hz) related spectral power of the same condition was also significantly lower compared to the activity related to the distribution of the ABA-triplets (A-B [approx. 10 Hz], [*t*(13) = −4.125, *p* < .*001*] and B-A [approx. 6 Hz], [*t*(13) = −4.312, *p* < .*001*]). Furthermore, the 2 Hz spectral power (B-tones) in the small frequency-separation condition (2-semitones) was significantly lower compared to the spectral power corresponding to A-B (approx. 10 Hz), (*t*(13) = −3.702, *p* < .*05*) and B-A tones (approx. 6 Hz) ,(*t*(13) = −8.068, *p* < .*001*). Regarding the 2-semitones condition, the corresponding activity to a separate A-stream perception at approx. 4 Hz was also significantly lower than the 10 Hz (*t*(13) = −3,320, *p* < .*001*) and 6.6 Hz (*t*(13) = 8,044, *p* < .*001*) spectral power. Similarly, concerning the intermediate frequency separation condition (4-semitones), *post hoc* comparisons revealed decreased activities corresponding to separate presentation of A-tones (approx. 4 Hz) and B-tones (approx. 2 Hz) as compared to spectral power of the A-B target frequency (approx. 10 Hz), (*t*(13) = −2.321, *p* < .*05*) and (*t*(13) = −2.321, *p* < .*05*), respectively.

In order to understand better the source of significant interactions from the previous 4 × 4 ANOVA and to explore the effect between the hemispheres, four additional ANOVAs were conducted wherein the mean spectral power of each target frequency was entered separately into repeated-measures 2 × 4 ANOVA using within-subject factors Hemisphere (left, right) and Conditions (0-, 2-, 4- and 10-semitones).

#### Activity related to a perception of separate A- and B-streams (parts 1 and 2)

The time-frequency outcome demonstrated that the 2 Hz activity (B-tones related) in the 10-semitones condition evolved at about 0.5 s and reached its maximum at about 0.8 s and 2 s. This was not so well pronounced in the case of 2- and 4-semitones and did not occur during 0-semitones condition (Figure 2AB, Table 1).

**Table 1:** Mean values of the baseline corrected spectral power across the conditions

**Frequency**	**Part-1**		**Part-2**		**Part-3**		
**RH**^**a**^	**0 ST**	**10 ST**	**2 ST**	**4 ST**	**A-stream**	**B stream**	**SA**^**c**^
10 Hz	3.615	5.377	12.448	12.790	- -	- -	- -
8 Hz	- -	- -	- -	- -	3.653	- -	−0.334
6.6 Hz	4.021	2.236	4.785	3.447	- -	- -	- -
4 Hz	1.375	1.680	2.352	7.210	- -	0.628	0.194
2 Hz	0.460	2.215	1.889	5.634	- -	- -	- -
**LH**^**b**^	**0 ST**	**10 ST**	**2 ST**	**4 ST**	**A-stream**	**B-stream**	**SA**^**c**^
10 Hz	1.392	−0.009	2.885	1.882	- -	- -	- -
8 Hz	- -	- -	- -	- -	4.576	- -	−0.822
6.6 Hz	3.102	0.597	2.455	1.979	- -	- -	- -
4 Hz	1.636	1.568	1.850	3.439	- -	1.475	0.416
2 Hz	0.613	3.942	0.084	3.253	- -	- -	- -

^**a**^**RH**-Right Hemisphere; ^**b**^**LH**-Left Hemisphere; ^**c**^**SA**- Baseline Corrected Spontaneous Brain Activity.

Regarding the 2 Hz activity (B-tones related), the ANOVAs revealed a significant difference between the conditions (main effect Condition [*F*(3,39) = 10.063, *p < .001*]). The following *Post hoc* pairwise comparisons showed that the activity at approx. 2 Hz (B-tones related rhythm) increased significantly with increasing the inter-tonal frequency separation between A- and B-tones. The 2 Hz-activity of the 10-semitones condition was significantly greater than in the 0-semitones condition (*t*(13) = −3.169, *p* < .*05*) and the 2-semitones condition (*t*(13) = 2.937, *p* < .*05*). The mean spectral power at 2 Hz of the 4-semitones condition was also significantly higher compared to the 2-semitones condition (*t*(13) = −3.967, *p* < .*05*) and the 0-semitones condition (*t*(13) = −3.934, *p* < .*05*). The comparison of the mean spectral power at 2 Hz, between the conditions with close inter-tonal frequency difference, did not reveal significance; 2-semitones vs. 0-semitones (*t*(13) = −0.812, *p* = .*431*) and 10-semitones vs. 4-semitones (*t*(13) = −1.901, *p* = .*080*).

As seen on the group level time-frequency plots, the 4 Hz-activity (A-tones related) showed greater enhancement in the second part (Figure 2B), wherein the frequency difference between the A- and B-tones was relatively small (2- and 4-semitones) when compared to the first part. The power of the signal at approximately 4 Hz was therefore more pronounced and better visible than at 2 Hz in the second part (Table 1, Figure 2B). The baseline corrected mean spectral power here differed significantly across conditions (main effect Conditions [*F*(3,39) = 6.115, *p < .05*]). *Post hoc* comparisons revealed that the spectral power at 4 Hz in case of 4-semitones was significantly greater than in case of 2-semitones condition (*t*(13) = -2.333, *p* < .*05*), the 10-semitones condition (*t*(13) = −2.709, *p* < .*05*) and the 0-semitones condition (*t*(13) = -2.618, *p* < .*05*). The 4 Hz-activity was also significantly greater in case of 2-semitones compared to t 10-semitones (*t*(13) = -2.173, *p* < .*05*).

**Figure 3:**
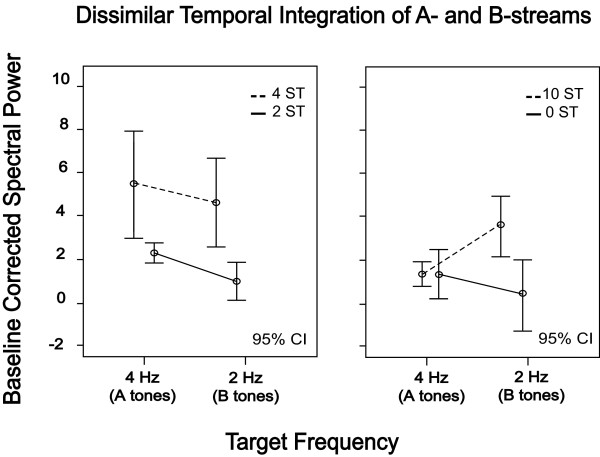
**Dissimilar efficiency of the temporal integration of segregated A (approx. 4 Hz) and B (approx. 2 Hz) streams.** The conditions from part 1 (0- vs. 10-semitones) and part 2 (2- vs 4-semitones) are presented on the right and on the left plot, respectively. Error bars indicate the 95 % confidence intervals for the within-subject effect (Condition x Target Frequency).

Figure 3 summarizes the differences of the spectral power related to the separate perception of the A-tones (4 Hz) and B-tones (2 Hz) of all conditions (0-, 2-, 4- and 10-semitones) across the participants. Error bars indicate the 95% confidence intervals for the within-subject effect (Condition x Target Frequency) [50].

#### Activity related to distribution of the tone intervals in the asymmetric ABA-triplets (part 1 and 2)

The activity corresponding to A-B and B-A intervals of the ABA-triplets (approx. 10 and 6 Hz) appeared to be sustained during the 0-semitones condition and transient in the conditions that allowed perceptual streaming (2-, 4- and 10-semitones), Figure 2AB.

The ANOVAs revealed that the mean values of the baseline corrected spectral power at around 10 Hz (related to the presentation rate of A-B segment in the ABA-triplet) in the first and second part did not change significantly between the conditions (Condition [*F*(3,39) = 2.588, *p = .067*]). The activity between the hemispheres, however, differed significantly (main effect Hemisphere [*F*(1,13) = 17.030, *p < .001*]). *Post hoc* pairwise comparisons showed that the spectral power at 10 Hz was generally greater in the Right Hemisphere (*RCSP = 3.427*) than in the Left Hemisphere (*RCSP = 1.037*), (*t*(13) = −4.281, *p* < .*001*).

Regarding the 6.6 Hz target frequency, the ANOVAs revealed significant main effect Condition (*F*(3,39) = 2.923, *p < .05*). The following *Post hoc* pairwise comparisons showed that the activity at approximately 6 Hz (B-A interval of ABA-rhythm) was significantly greater in the case of 2-semitones compared to 10-semitones *t*(13) = 2.474, *p* < .*05*). There were no other significant comparisons in the 8 Hz target frequency (0- vs. 10-st. [*t*(13) = 1.954, *p* = .*073*], 2- vs 4-st. [*t*(13) = 1.293, *p* = .*219*], 4- vs. 10-st [*t*(13) = 1.705, *p* = .*112*], 0- vs. 4-st. [*t*(13) = .884, *p* = .*178*] and 0- vs. 2-st [*t*(13) = −.156, *p* = .*878*]).

#### Activity related to the independent presentation of A- and B-tones (part 3)

Unlike the ABA-structure from the first two parts this scenario could not provide two alternative perceptual states (integrated vs. segregated). Therefore, the participants were not required to pay attention to the ongoing presentation. The auditory system prefers regular arrangements [39,40] and hence two steady-state responses corresponding to presentation rates of the two sequences were expected in the time-frequency spectrum. As shown at the plots (Figure 2C), the non-attended condition (part 3) elicited an activity enhancement at about 8 Hz and 4 Hz, corresponding to the independent A- and B-tones presentation rates. The baseline corrected mean spectral power values of the third part vs. the Spontaneous activity at 8 Hz and 4 Hz, across hemispheres, are shown in Table 1.

The ANOVAs showed a significant difference between the different activities (main effect Activity [*F*(3,39) = 12.759, *p < .001*]). *Post hoc* pairwise comparisons revealed that the spectral power corresponding to an independent presentation of A- and B-streams (approx. 8 Hz and 4 Hz, *RCSP = 9.595*) during the stimulation was significantly greater than the spectral power at 8 Hz and 4 Hz during the resting state measures (*RCSP = −.852*), (*t*(13) = 5.207, *p* < .*001*).

### Source waveform data

**Figure 4:**
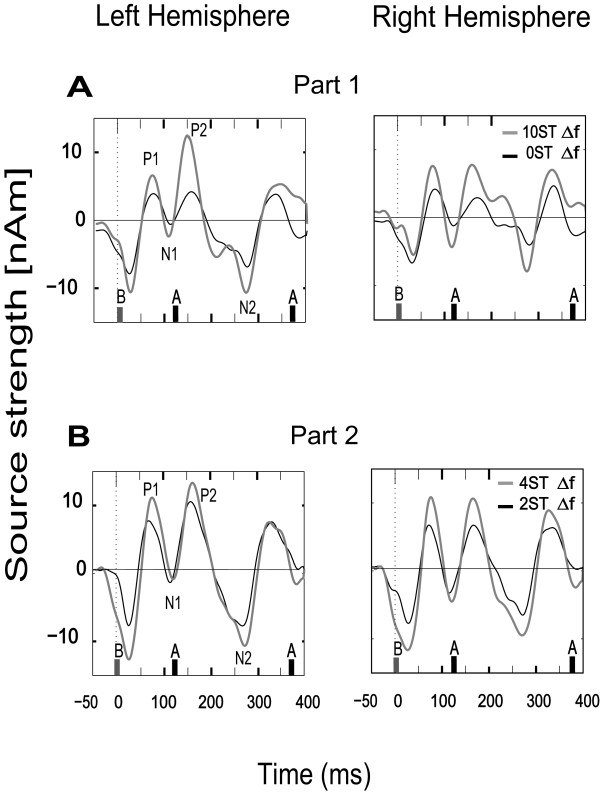
**Grand averaged source-waveforms triggered to B-tones of the asymmetric ABA-triplets.** The data-from Left and Right Hemispheres are plotted as Source Strength (nAm) against Time (ms). **(A)** Two different degrees of frequency separation between A- and B-tones (Δf = 0-semitones [black lines] and Δf = 10-semitones [grey lines]) which were presented in the first experimental part are shown. The 0-semitone condition refers to the non-streaming condition and 10-semitones to the streaming condition. **(B)** The second experimental part presented stimuli of Δf = 2 (black lines) and Δf = 4-semitones (grey lines), referring to small and intermediate Δf-conditions.

Clearly identifiable evoked responses were obtained from all subjects. The magnitude of the P1 component of the responses to B-tones of the ABA-triplets raised with increasing the frequency separation (significant main effect of Condition [*F*(3,33) = 7.386, *p* < .*001*]), Figure 4AB. *Post hoc* comparisons revealed that the magnitude of the P1 component across the trials was significantly greater in the 10-semitones condition than in the 0-semitones (*t*(11) = -3.387, *p* < .*05*) and 4-semitones conditions (*t*(11) = 2.474, *p* < .*05*). Additionally, the amplitude was significantly greater in the case of 4-semitones than in the 2-semitones (*t*(11) = −3.101, *p* < .*05*) and 0-semitones (*t*(11) = −3.720, *p* < .*05*). There were no other significant comparisons regarding the P1 amplitude (0- vs. 2-st. [*t*(11) = 1.966, *p* = .*075*] and 2- vs 10-st. [*t*(11) = 0.457, *p* = .*656*]).

The N1 component also increased following the increased frequency separation, however not significantly (Condition [*F*(1,11) = 1.435, *p = .250*]). The two components did not show any effects or interactions concerning the hemispheres: P1-effect Hemisphere (*F*(3,33) = .164, *p = .694*) and N1-effect Hemisphere (*F*(1,11) = .111, *p = .746*). Despite the fact that P2 and N2 were not entered into statistical analyses, it should be noted that they appeared to be enhanced in the case of streaming and intermediate ∆f, compared to single-stream and small with the lowest frequency limit of 1 Hz.∆f conditions, respectively (Figure 4AB). These components likely represent the activity related to the second A-tone of the ABA-triplet.

## Discussion

The present study combines time-frequency analysis on a sensor space level with source waveform analysis by means of magnetoencephalography (MEG) to explore the underlying neural activity behind the processing of an ABA-triplet streaming-task. We furthermore challenge the perception by contrasting four degrees of inter-tonal frequency separation and thus enabling the formation of different perceptual states in one and the same polyrhythmic structure. In order to keep sustained attention, the participants were instructed to focus on the slowest rhythm (B-tones). The results of the first two parts (presentation of asymmetric ABA-triplet sequences) revealed a clear increase of the spectral power at approximately 2 Hz that corresponds to the B-tones presentation rate in the streaming (10-semitones) and intermediate frequency separation (4-semitones) conditions. This was in line with our hypothesis. Additionally, the A-tones presentation rate elicited steady-state like activity at approximately 4 Hz. The ABA-triplet sequence used in the present study is usually heard as a galloping rhythm and the A- and B-streams are enclosed into the ABA-pattern [38]. Hence the A- and B-tones related activities at 4 Hz and 2 Hz are only accessible in the spectrum if the two streams are segregated. Our results, therefore, likely reflect the selective segregation of the polyrhythmic ABA-pattern into two monorhythmic A- and B- streams. The activity at approximately 10 Hz and 6 Hz that corresponds to A-B and B-A-tone intervals of the ABA-triplets also increased across the trials in the first two parts. In the light of the present findings, one might speculate that the neural representation of different auditory sequences relies on neural entrainment of the temporal intervals between the composed stimuli. Therefore, when the perception is in favor of one-stream condition (0-semitones) one could capture the corresponding presentation rates in the spectrum (10 Hz and 6.6 Hz), whereas the other rhythms would be suppressed (2 Hz and 4 Hz) and vice versa in the case of segregation (10-semitones). Additionally, the time-frequency results demonstrated that the responses to the ABA-frequency distribution (approx.10 Hz and 6.6 Hz) appeared to be sustained across the entire presentation of the non-streaming condition (0-semitones), whereas the B-tone related activity (2 Hz) emerged at approximately 0.5 s and reached its maxima at approx. 0.8 s and 2 s only during the streaming condition (10-semitones). Conversely, the spectral power at 10 Hz and 6.6 Hz was rather transient in all other conditions that allowed perceptual streaming (2-, 4- and 10-semitones). The streaming phenomenon is cumulative [51] and needs variable amount of time to build-up [16] and therefore the appearance of the 2 Hz activity at about 0.5 s in the time-frequency plots likely reflects the streaming built-up period. Alongside this, the vanishing of the activity at approx. 10 Hz and 6 Hz could match the periods wherein the perception alternated in favor of stream segregation. Indeed, the statistical analysis revealed that the spectral power corresponding to the A-B and B-A time intervals of the ABA-triplets is significantly enhanced compared to the responses tuned to the separated A- and B-tones in the non-streaming scenario (0-semitones) and the conditions of small and intermediate inter-tonal frequency separations (2- and 4-semitones).

The statistical analysis showed furthermore that the steady-state activity related to the attended B-stream (2 Hz) increased significantly with enlarging the inter-tonal frequency difference between A- and B-tones (from 0- to 10-semitones). This result lends further support to the idea that attention is a crucial factor in auditory streaming because it biases the auditory system towards particular grouping or binding of sound-source elements in favor of the listener’s intention [19,21]. A previous study by Xiang and colleagues, for instance, explored the mechanisms of temporal integration and its interaction with attention in the auditory system by using a streaming paradigm with two competing tones [20]. The authors demonstrated that focusing the listeners’ attention on one of the two competing tempi enhances significantly its steady-state power. However, the two competing tones they used could primarily produce two auditory streams [20], unlike the asymmetric ABA-triplets used in the present study. Furthermore, it has been demonstrated previously that the steady-state responses could be modulated by attention [35,36]. Our experimental design, therefore, allowed us to explore the interaction between the temporal rates in one integrated polyrhythmic pattern and two segregated monorhythmic streams in one and the same tone-sequence. On the other hand, our results revealed a higher spectral power tuned to the A-tones presentation rate (4 Hz) in comparison with the B-tones related responses (2 Hz) in the cases of intermediate and small frequency separation between tones, although the attention was focused on the B-rhythm. It might be suggested that in cases of small frequency differences between tones, such as those used in the second part (2- and 4-semitones), the perception of the B-tone is not able to dominate the perception of the A-tones, and that this produces considerably higher activity at approximately 4 Hz target frequency. It could be speculated therefore, that a greater effort is needed to segregate the ABA-structure onto separate A- and B-tone streams in the cases of small and intermediate frequency differences than in the pure streaming condition (10-semitones). In addition, it might be more difficult to follow the slower B-stream (2 Hz) instead of the twice as fast A-stream (4 Hz) in the cases of intermediate and small frequency separations than in the greater frequency differences. Besides that, previous studies showed that the steady-state responses are stronger in low frequency rates (below 16 Hz) when mediated by attention [21,52]. Although the attention was focused on the B-tones in our experiment, changing the inter-tonal frequency separation into the ABA-tone pattern revealed dissimilar efficiency of temporal integration of separate A- and B-streams. It has been demonstrated previously that the P1 and N1 components of the human AEFs are larger when listeners perceive two segregated streams than one integrated stream and this magnitude augmentation is consistent with the increasing frequency separation between the A- and B-tones [2]. However, these authors showed that the B-tones’ related responses were always enhanced, regardless of the attended stream (A- or B-tones) [2]. Similarly, it has been proposed that the frequency separation between different sound sources of a polyrhythmic sequence is sufficient to provide the selective processing of a particular musical instrument; however, the selective attention to one or another spatially separated element of this rhythm could additionally improve the segregation process [53]. These findings together support the idea that the attention in auditory streaming is not merely an intrinsic mechanism that augments the neural responses but its effects are based on a specific interaction between the physical attributes of the stimuli [20]. Additionally, the present outcome is in line with the hypothesis that distinct neuronal populations are involved in the processing of A- and B-tones and suppression of one population might underlie the stream segregation phenomenon [11,12].

Assuming that the steady-state activity at low frequency bands is generated by the periodic appearance of the evoked components in response to the A- and B-tones, we tested whether the source waveform of the response signal triggered by the attended B-tones of the ABA-triplets represents any significant effects regarding the evoked peaks. Moreover, the modulation of the source waveforms’ components synchronized to each triplet of the ABA-streaming task is a traditional way to investigate the auditory streaming phenomenon (see e.g. [2]). The analysis revealed higher amplitude of the evoked components with increasing the frequency separation, a finding that is in line with prior studies [2,6,8,54-56]. Specifically, the P1 evoked component to the B-tones enhanced significantly as the inter-tonal frequency difference increased. This implies that the enhancement of the evoked fields in the source space level together with the B-tones related activity derived from the time-frequency results likely reflect the selective segregation of the attended B-stream. However, the source-wave forms comprise more than one harmonics in the spectrum and it is thus difficult to separate the streaming-related effects from the activities related to the physical features of the sounds. Elhilali and colleagues, for instance, demonstrated that frequency-distant spectral components are no longer heard as separate streams if presented synchronously rather than consecutively, while the neural activity increases with increasing frequency separation between tones [18].

Hence, the auditory evoked fields per se are not capable of fully explaining the perception of streaming.

In apparent contrast to the first two parts, two recurring A- and B-tone-streams were presented in the third part. Here, the temporal distribution between the A-B and B-A-tones was always different, whereas the presentation rates of the A-tones and the B-tones per se, were always regular, corresponding to 8 Hz and 4 Hz, respectively. The results demonstrated clear non-attentive steady-state activity at approx. 8 Hz and 4 Hz. Indeed, it has been shown that the auditory system prefers regular arrangements [39,40]. Moreover, the integration of auditory streams, based on their regularities, could take place automatically. The mismatch negativity component (MMN) of event-related potentials, for instance, automatically detects changes in the regular stimulus pattern [57-60]. Additionally, it has been found that the MMN operates also on the basis of auditory objects and that the integration of objects occurs pre-attentively in the auditory system [61]. The experimental design applied in the third part could not provide two complementary percepts (integrated vs. segregated), such as ABA-triplets. It could be speculated, therefore, that the two auditory streams were formed of the very first moment of their presentation. On the other hand, it has recently been demonstrated that stream-integration can occur with irregular arrangements [42]; however, it is likely that in the absence of active awareness, the auditory system integrates tone patterns based on their physical regularities.

In summary, the present findings suggest that neural encoding of a streaming task relies on an oscillatory entrainment of the stimulus presentation rates. However, two separate effects of the time-frequency data must be distinguished: the first is represented in our results by the distribution of the intervals between the A-B (10 Hz) and B-A (6.6 Hz) tones of the ABA-triplets (0-semitones). The second effect is represented by the 2 Hz and 4 Hz steady-state responses related to the B- and A-tones derived from the conditions that allow perceptual streaming (2-, 4- and 10-semitones), alongside the steady-state effects of non-attentive listening (part 3). The present effects cannot be directly ascribed to the underlying mechanisms responsible for various perceptual states, because the participants were not required to make streaming judgments during the trials. Nevertheless, these effects might be grounded to physiological hallmarks of the process, which precedes the formation of one vs. two streams percept. Hence, further study is necessary to show the differences in the spectral distribution of identical tonal-frequency separation in conditions of perceptual validation during integration vs. segregation.

## Conclusions

The present findings are consistent with previous studies suggesting that the perceptual organization in sequential auditory scene analysis relies on oscillatory entrainment to task-driven sound input. The results showed that increasing of the frequency separation between A- and B-tones of the ABA-pattern correlates with a greater magnitude of the steady-state responses tuned to the attended B-tones. Alongside this, the P1 evoked fields’ component, synchronized to the B-tones, increased in amplitude with raising the inter-tonal frequency difference. Furthermore, the asymmetric ABA-objects, spontaneously elicited sustained activity, which corresponded to the temporal distribution of the constituent tone intervals. The results also revealed that the efficiency of temporal integration of separate streams is dissimilar depending on the degree of frequency separation between the competing sounds. The steady-state responses tuned to the B-tones dominated the responses tuned to the A-tones in the case of great frequency difference between tones. Conversely, the representation of the A-tones dominated the B-tones in the cases of small and intermediate frequency separation, in which the task required greater effort. Overall, the present outcome suggests that the neural effects of auditory stream integration and segregation could be directly captured in the time-frequency spectrum and measured with significance tests at the sensor space level.

## Competing interests

The authors declare that they have no financial or any other competing interests.

## Authors’ contributions

IC, RD and CP conceived of the study designed the experimental setup and the auditory stimuli. IC acquired the data. IC performed the data & statistical analyses. All authors participated in the data evaluation and interpretation and in writing the manuscript, and have approved the final version of the manuscript.

## Supplementary Material

Additional file 1 Two separated plots show the Grand Averaged topographic Maps (A) and the neural responses of one Representative Subject (B). Click here for file
