# Supplementary material for: Perceptual organization of auditory streaming-task relies on neural entrainment of the stimulus-presentation rate: MEG evidence
Source: BMC Neurosci. 2013 Oct 12;14:120. doi: 10.1186/1471-2202-14-120 (PMC3853018; doi:10.1186/1471-2202-14-120)
Supplement: Additional file 1 — Two separated plots show the Grand Averaged topographic Maps (A) and the neural responses of one Representative Subject (B). [file 1471-2202-14-120-S1.pdf]

Supplemental information

Perceptual organization of auditory streaming-task relies on neural entrainment of the stimulus-presentation rate: MEG evidence

Ivan Chakalov[1, 2]*, Rossitza Draganova[2]*, Andreas Wollbrink[1], Hubert Preissl[2], Christo Pantev[1§]

Inventory of Supplemental Information

Additional File 1A (Topographic Maps)

Additional File 1B (Representative Subject)

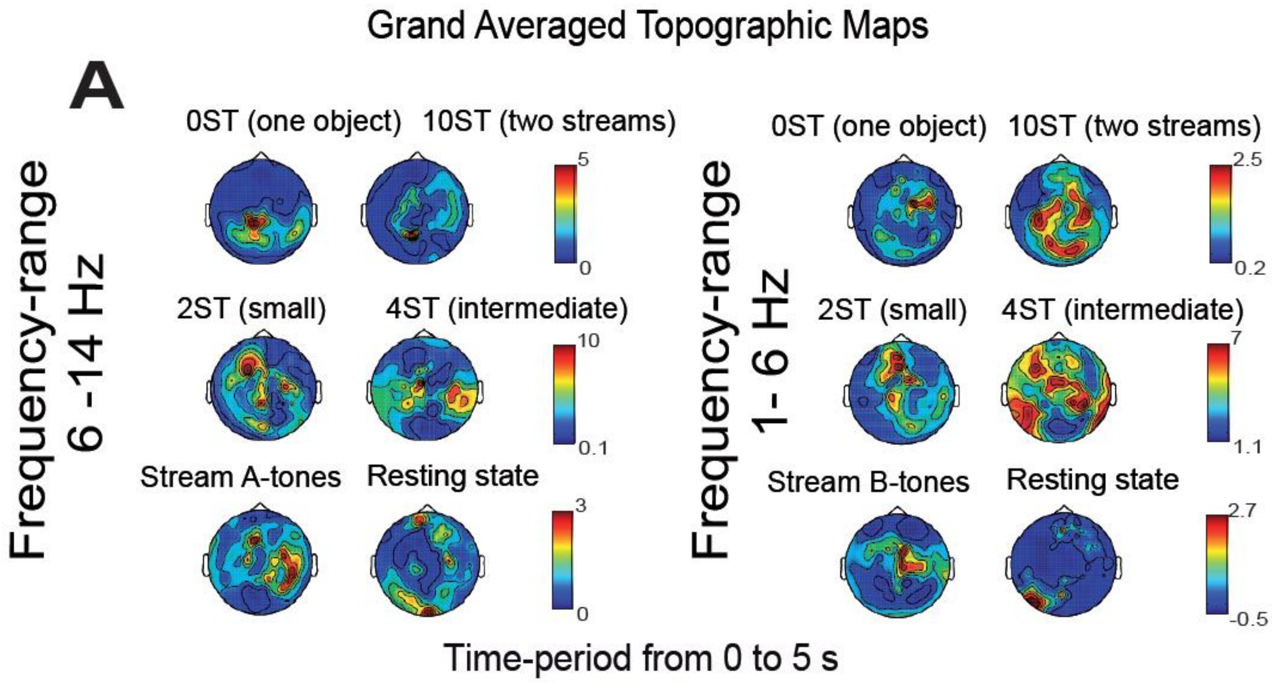

Additional File 1 (A) Grand averaged topographic maps depicting the time course of each condition (from 1 to 5 s). The left and right panels represent the responses across conditions in frequency ranges of 6 to 14 Hz and 1 to 6 Hz, respectively. ***Part 1 (upper block)***: non-streaming versus streaming conditions. ***Part 2 (middle block)***: small vs. intermediate Δf conditions ***Part 3 (bottom block)*****:** independent presentation of A- and B-sound streams and the relevant spontaneous activity in the same frequency bands.

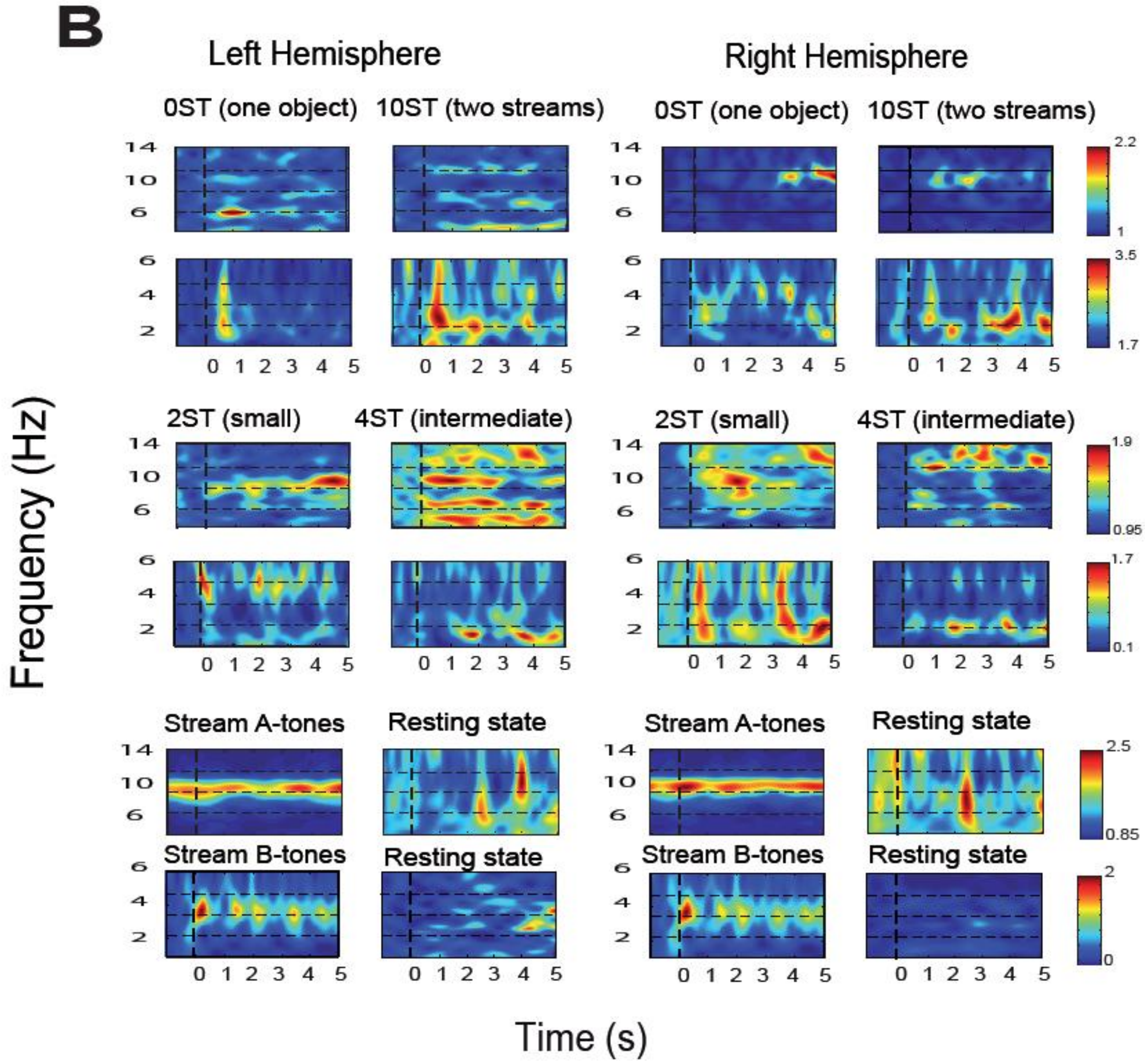

Additional File 1 (B) Individual level time-frequency plots from one representative participant. The data were averaged across all trials and pre-selected MEG channels separately for the Left (LH) and Right (RH) Hemispheres. Upper plots in each panel represent the frequency range from 6 to 14 Hz; lower plots the frequency range from 0 to 6 Hz. The bands are presented separately for the LH and RH. Color bars represent the relative change of the spectral power. The strength of the signal is color-coded; high strength is denoted with red and low strength with blue. ***Part 1 (top panel)***. Non-streaming versus streaming conditions are presented independently for LH and RH. ***Part 2 (middle panel)***. Small vs. intermediate Δf conditions are presented in the same way as Part 1. ***Part 3 (bottom panel).*** The plots show the neural effects of simultaneous presentation of independent A- and B-sound streams and the relevant resting brain-state.
